# Degradation Product-Promoted Depolymerization Strategy for Chemical Recycling of Poly(bisphenol A carbonate)

**DOI:** 10.3390/molecules29030640

**Published:** 2024-01-30

**Authors:** Maoqing Chai, Guangqiang Xu, Rulin Yang, Hongguang Sun, Qinggang Wang

**Affiliations:** 1 College of Polymer Science and Engineering, Qingdao University of Science and Technology, Qingdao 266042, China; chaimq@qibebt.ac.cn; 2 Key Laboratory of Biobased Materials, Qingdao Institute of Bioenergy and Bioprocess Technology, Chinese Academy of Sciences, Qingdao 266101, China; yangrl@qibebt.ac.cn; 3 Center of Materials Science and Optoelectronics Engineering, University of Chinese Academy of Sciences, Beijing 100049, China; 4 Shandong Energy Institute, Qingdao 266101, China; 5 Qingdao New Energy Shandong Laboratory, Qingdao 266101, China

**Keywords:** PC, bisphenol A, plastic wastes, depolymerization, chemical recycling

## Abstract

The accumulation of waste plastics has a severe impact on the environment, and therefore, the development of efficient chemical recycling methods has become an extremely important task. In this regard, a new strategy of degradation product-promoted depolymerization process was proposed. Using *N*,*N*′-dimethyl-ethylenediamine (DMEDA) as a depolymerization reagent, an efficient chemical recycling of poly(bisphenol A carbonate) (BPA-PC or PC) material was achieved under mild conditions. The degradation product 1,3-dimethyl-2-imidazolidinone (DMI) was proven to be a critical factor in facilitating the depolymerization process. This strategy does not require catalysts or auxiliary solvents, making it a truly green process. This method improves the recycling efficiency of PC and promotes the development of plastic reutilization.

## 1. Introduction

Plastics are essential materials in our daily lives and are used for various purposes [1]. However, the accumulation of waste plastics, caused by improper use and indiscriminate disposal, is having a serious impact on the environment. Hence, it is necessary to develop an environmentally sustainable plastics economy that recycles these materials at the end of their life cycle [2,3,4,5,6]. Currently, the main methods of plastic recycling are categorized into physical and chemical recycling. Physical recycling is a downcycling mode due to the thermo-mechanical degradation caused by the harsh melt conditions, which ultimately prevents the material from being reused in the manufacture of high-end products [7,8,9]. In contrast, chemical recycling can convert waste plastics into initial monomers or high-value chemicals for upcycling while combining multiple advantages such as resource utilization, environmental friendliness, quality control, and economic benefits [10,11,12,13,14]. Therefore, the development of efficient chemical recycling strategies has become an extremely important task.

In this regard, researchers have made great efforts in recent years. For example, Zhang et al. proposed a biomimetic catalyst strategy to promote depolymerization. They designed a dual-core zinc catalyst with biomimetic Zn-Zn sites that activate the plastic, stabilize key intermediates, and enable intramolecular hydrolysis. This catalyst was stable over a wide range of operating temperatures (30–340 °C) and pH values (8–14), allowing for sustainable recycling of poly (ethylene terephthalate) (PET) [15]. Moreover, Odelius et al. reported a solvent-facilitated depolymerization strategy. The reduced *T*_c_ was observed by selecting a suitable solvent, such as dimethylformamide, that strongly interacts with the monomer, which allowed the high-molecular-weight poly(L-lactide) to be directly chemically recycled to L-lactide within 1–4 h at 140 °C, with a reaction selectivity of up to 98–99% [16]. Recently, Wang’s team established a new method to accelerate mass transfer depolymerization by mixing solvents. Complete hydrolysis of unsaturated polyester resin (UPR) was achieved by selective cleavage of ester bonds. Under the synergistic effect of tetrahydrofuran (THF) and H_2_O, the depolymerization could be carried out under conditions at 100 °C [17]. In addition, strategies that drive polymerization–depolymerization to proceed rapidly at low energies by designing systems in thermodynamic near-equilibrium have also received attention [18,19,20,21]. For example, Lu et al. designed a thermodynamically neutral system based on β-thiolactones by introducing a gem-dimethyl group on a four-membered ring. The obtained polythioesters (PTEs) could be rapidly recycled into pristine, enantiomerically pure β-thiolactones at room temperature with low energy input [22]. Despite these advances, there is still an urgent need to develop more types of depolymerization strategies, given the diversity of plastics and the complexity of the practical realities of degradation. These strategies greatly promote the development and progress of the plastic recycling field. However, it is especially necessary to develop novel strategies while reducing or avoiding the use of catalysts, solvents, or depolymerizing reagents to achieve green depolymerization methods.

Our research group has been committed to the study of chemical depolymerization strategies. For example, a “DE-RE polymerization” strategy was proposed to achieve the recycling process from waste poly(lactide) (PLA) plastic to new virgin-quality PLA materials in a “polymer-to-polymer” mode [23,24]. The selective depolymerization of various mixed plastics was achieved by sequential or “one-pot” depolymerization strategies to obtain the initial monomers or value-added chemicals [25]. To further expand the research field of plastic depolymerization, in this study, we proposed a new degradation product-promoted depolymerization strategy. N,N′-dimethyl-ethylenediamine (DMEDA) was used as a depolymerizing agent to efficiently convert discarded PC material into the original monomer, bisphenol A (BPA), and a value-added chemical, 1,3-dimethyl-2-imidazolidinone (DMI), under the promotion of 1 equivalent of DMI. The promoting effect of the product DMI on depolymerization was systematically studied. It is noteworthy that various commercial PC plastics could be applied to this strategy, as well as to the selective depolymerization of PC/ABS and PC/PET mixed plastics. This strategy provides a new idea and method for solving the recycling problem of PC.

## 2. Results and Discussion

### 2.1. Depolymerization of PC under Solvent-Free Conditions

In our previous work, amino-alcoholysis of PC materials under solvent-free and catalyst-free conditions was achieved with 1 equiv. of amino alcohol utilizing the nucleophilicity of the depolymerizing reagent [26]. This success motivated us to further investigate the ammonolysis of PC materials, in which a higher nucleophilicity of amine may lead to milder reaction conditions. Therefore, in this paper, *N*,*N*′-dimethyl-ethylenediamine (DMEDA), a common diamine compound, was chosen as the depolymerizing agent. To minimize energy consumption, the solvent-free ammonolysis experiments on PC were conducted at a mild temperature of 80 °C.

**Figure 1:**
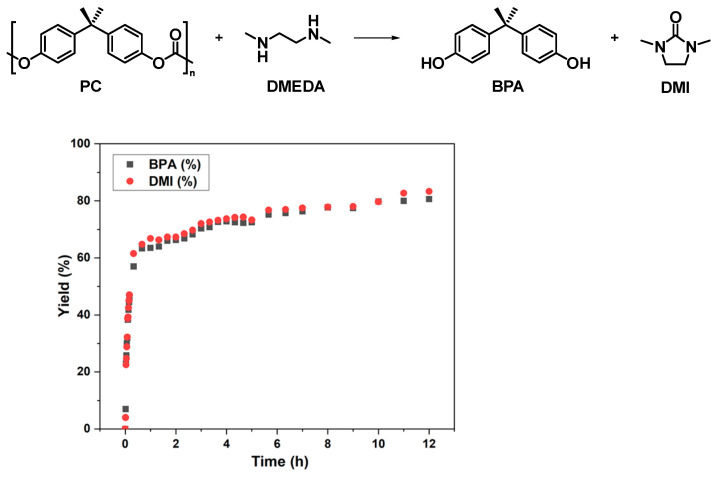
Degradation of PC under solvent-free conditions at 80 °C: PC pellets (sizes: 3–4 mm, *M*_n_ = 23.8 kg/mol, *Đ* = 2.78, 508 mg, 2 mmol based on BPA unit), *N*,*N*′-Dimethyl-1,2-ethanediamine (DMEDA) (229 μL, 2.12 mmol), acetic acid as quencher, and dibromomethane (140 μL, 2 mmol) as an internal standard to calculate yields.

As shown in Figure 1, it was found that the depolymerization proceeded rapidly at the beginning, providing bisphenol A (BPA) and 1,3-dimethyl-2-imidazolidinone (DMI) in 73% and 74% yields, respectively, after 4 h. However, with the subsequent extension of the reaction time, the yields of degradation products hardly increased. The limit of yields could only be maintained at about 70–80%, suggesting that an equilibrium state had been reached. Upon analyzing the ^1^H NMR spectrum of the reaction at 24 h (Figure S1: 24 h, BPA 80%, DMI 82%), it was found that the remaining DMEDA was still present in the system, but it no longer underwent depolymerization with PC.

**Figure 2:**
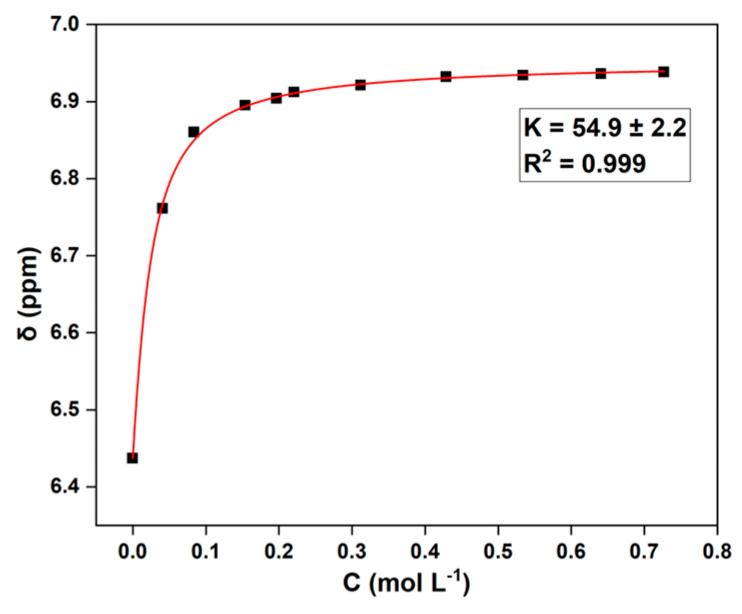
Determination of binding constant: BPA and *N*,*N*′-dimethyl-1,2-ethanediamine (DMEDA).

The unexpected phenomenon of PC being difficult to fully convert drove us to further investigate the underlying reasons. Compared to ethanolamine (amino alcohol), the depolymerization reagent DMEDA (diamine) showed a stronger basicity. From this feature, we deduced that the amino groups of the feedstock DMEDA may form strong hydrogen bonding with the phenolic hydroxyl groups of the product BPA, especially when a large amount of BPA is generated in the late stage of the reaction, thus hindering the smooth progress of depolymerization. To verify this hypothesis, the binding constant (K) between BPA and DMEDA through the O-H···N interaction was determined using concentration variation experiments with ^1^H NMR titration [27,28]. As shown in Figure 2, a high value of K was calculated as 54.9 ± 2.2, which was significantly greater than the O-H···O hydrogen-bonding constants (K = 0.9–10.9) reported for a series of hydroxylated biomass compounds [29,30]. This result indicated that there was a strong interaction between BPA and DMEDA. When they were tightly bound together, the amine group of DMEDA could not be exposed to attack the carbonyl group of the polymer chains, resulting in the PC material not being completely depolymerized.

### 2.2. Effect of Solvent Type on the Reaction

Next, to achieve complete conversion of PC, the influence of adding an auxiliary solvent on the reaction was investigated (Table 1). A series of solvents with good solubility for PC, such as dichloromethane (DCM) and tetrahydrofuran (THF), were prioritized. However, even with an extended reaction time of 24 h and solvent assistance, the yield of BPA was only 77% and 68%, respectively (entries 1, 2). In the poor solvent case, similar degradation results were observed when ethyl acetate (EtOAc), toluene (Tol.), and acetone (Ace.) were employed, with BPA yields of 70–75% indicating incomplete conversion as well (entries 3, 4, 5). It is worth noting that the product DMI could be used as a solvent in various organic synthesis and transformations; thus, it was also taken into account. Surprisingly, when using DMI as the solvent, a high yield of 87% for BPA was reached, showing that the previous reaction limit was broken (entry 6). Surprisingly, by monitoring the reaction process, a significant amount of unclosed products was identified at the early stage of the reaction in the case of other solvents, while these products were not obvious in DMI (entries 1–5, Figure S3). This phenomenon indicated that the ring-closing rate was also accelerated in DMI (Figure S4). Among all the results mentioned above, it was found that DMI performed obviously better than other solvents and had a favorable promoting effect on depolymerization.

### 2.3. Probe into the Role of DMI

**Figure 3:**
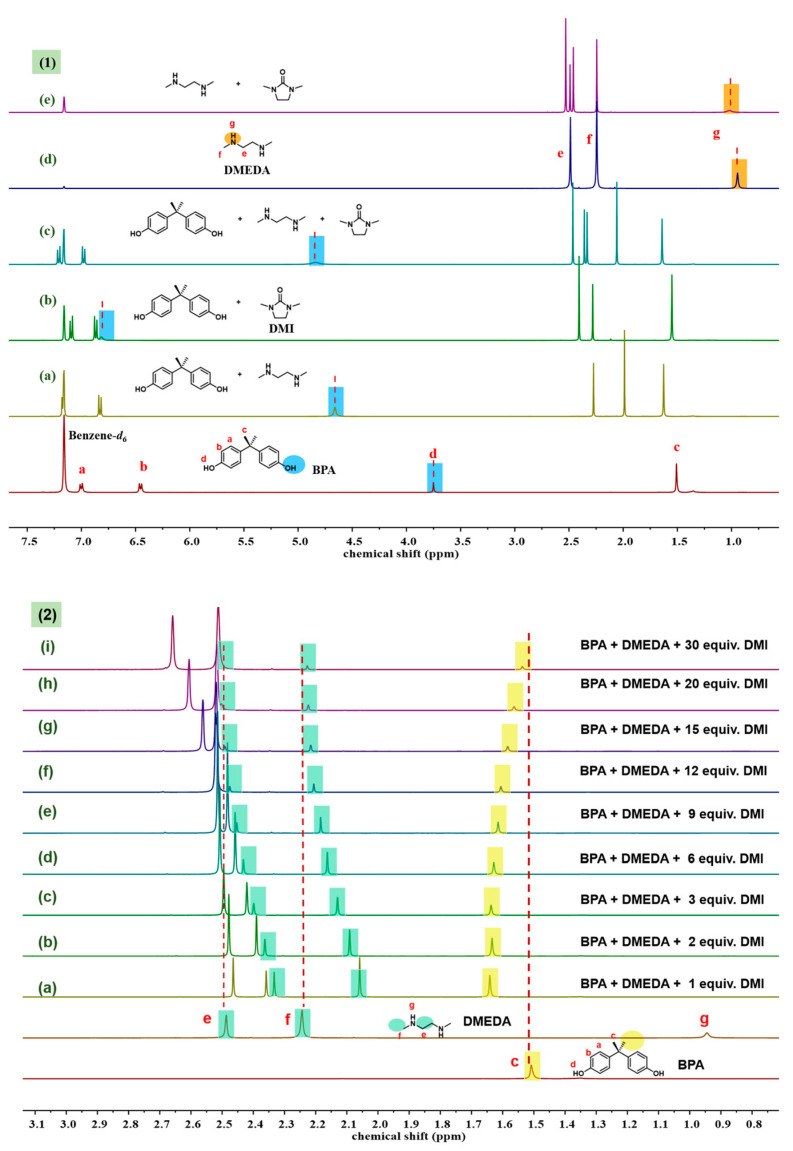
^1^H NMR overlapped spectra of chemical shifts of BPA, DMEDA, and DMI at different DMI concentrations in benzene-*d*_6_. (**1**) The chemical shift changes of active hydrogen, such as the hydroxyl groups of BPA marked with blue shading (**a**–**c**); the amino groups of DMEDA are marked with orange shading (**d**,**e**). (**2**) The chemical shift changes of the methyl and methylene parts, (**a**–**i**) show the changes in the methyl and methylene chemical shifts of DMEDA when the dosage of DMI increases from 1 equivalent to 30 equivalent, marked with green shading; the changes in the chemical shift of the methyl portion of BPA are marked with yellow shading.

After finding that DMI had an outstanding effect, a series of ^1^H NMR experiments were conducted to investigate the role of DMI during depolymerization. Deuterated benzene was used as a weak hydrogen-bond acceptor solvent to minimize the effects of deuterated solvents. From Figure 3(1), it can be observed that when BPA was mixed in a 1:1 molar ratio with DMEDA, the chemical shift of the -OH proton in BPA was downfield. For example, the chemical shift of the phenol hydroxyl proton of free BPA was 3.75 ppm, while it shifted to 4.65 ppm when interacting with DMEDA (Figure 3(1a)). It is well known that hydrogen bonding causes the depletion of electron density around the proton and the de-shielding of the nucleus; thus, protons participating in hydrogen bonding display a low-field shift in resonance frequency [31]. The change in chemical shift further proved the existence of hydrogen-bond interactions between BPA and DMEDA, as mentioned before. When 1 equiv. of DMI was added to BPA and the BPA−DMEDA system, the chemical shifts of the phenol hydroxyl proton of BPA shifted to 6.81 ppm and 4.83 ppm, respectively (Figure 3(1b,c)). This could be explained by the carbonyl oxygen of the amide portion of DMI, which acts as a good proton acceptor for BPA and DMEDA, thus leading to the chemical shift change in the hydroxyl proton. Additionally, when DMI was added, the chemical shift of the proton on the DMEDA amino group was also observed to change from 0.94 ppm to 1.02 ppm, which proved that DMI readily forms hydrogen bonds with DMEDA (Figure 3(1d,e)). The results above indicate that the addition of DMI changed the original hydrogen-bonding network in the system.

In addition to the significant changes in the chemical shifts of the active hydrogen protons of BPA and DMEDA, Figure 3(2) also shows that the chemical shifts of the -CH_3_ proton of BPA and the -CH_2_ and -CH_3_ protons of DMEDA underwent considerable changes. When 1 equiv. of DMI was added to the BPA-DMEDA system with a molar ratio of 1:1, the Δδ value of the -CH_3_ proton of BPA was 0.13 (Figure 3(2a)). As the amount of DMI gradually increased to 30 equiv., the Δδ value decreased to 0.02 (Figure 3(2i)). Similarly, with increasing amounts of DMI, the chemical shift changes in DMEDA also weakened. The Δδ value of the -CH_3_ proton on DMEDA decreased from 0.19 to 0.01. When the amount of DMI was 30 equiv., the chemical shifts in BPA and DMEDA almost returned to their initial positions, similar to their free states. These results indicate that DMEDA and BPA were released when DMI disrupted the hydrogen-bonding interaction between DMEDA and BPA.

**Figure 4:**
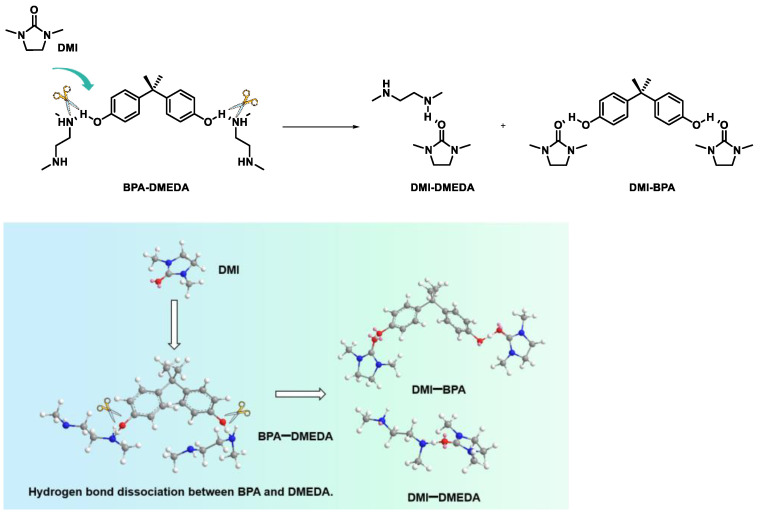
Schematic diagram of H-bond breaking after adding DMI molecules to BPA and DMEDA system.

From the results above, a possible reaction mechanism for product-promoted depolymerization was proposed. The amino group of DMEDA nucleophilically attacked the carbon atom of the carbonyl group, yielding the intermediate monoaminocarbamate. Subsequently, due to the strong polarity of the added product DMI, the intermediate underwent an intramolecular cyclization pathway to form DMI and BPA. This step was very rapid, with little occurrence of oligomeric intermediates. On the other hand, DMI had a high donor number (DN) and acted as a strong electron donor (Table S1). The carbonyl oxygen in DMI was an excellent hydrogen-bond acceptor, which disrupted the hydrogen-bonding network between BPA and DMEDA in the system, causing DMEDA to exist in its free state. This allowed the ammonolysis reaction of PC to proceed completely (Figure 4).

### 2.4. Optimization of DMI Equivalent

**Figure 5:**
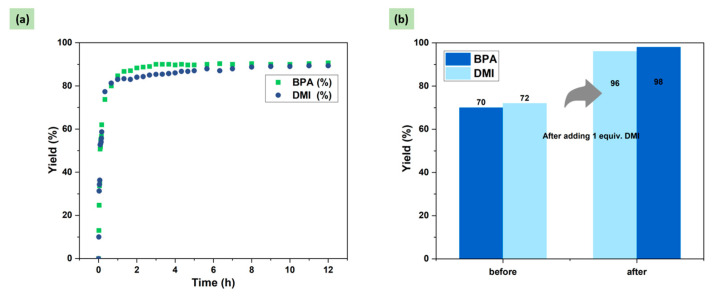
(**a**) Degradation of PC under conditions involving 1 equiv. of DMI at 80 °C: PC pellets (508 mg, 2 mmol based on BPA unit), *N*,*N*′-dimethyl-1,2-ethanediamine (DMEDA) (229 μL, 2.12 mmol), dibromomethane (140 μL, 2 mmol) as an internal standard, and acetic acid as quencher. (**b**) Depolymerization of PC by DMEDA under solvent-free conditions and after adding 1 equiv. of DMI.

After clarifying the mechanism of DMI, DMI additions were further reduced to improve the economics of the reaction process. The addition of DMI was adjusted to 1 equiv., and the depolymerization process was monitored as well. As shown in Figure 5a, with the addition of only 1 equiv. of DMI, the reaction process was significantly promoted compared with the process without the addition (Figure 1), and the final product yield increased from 70% to 90%. This result indicated that the addition of 1 equiv. of DMI was sufficient to break the original upper limit of the reaction and promote the reaction to some extent. In addition, as shown in Figure 5b, the yields of BPA and DMI were 70% and 72%, respectively, for 3 h of reaction under solvent-free conditions, and after continuing the reaction for 2 h, the yields of the two products remained almost unchanged (72% and 73%, respectively). However, when 1 equiv. of DMI was added to the solvent-free system after 3 h of reaction and allowed for an additional 2 h of reaction, complete degradation of PC was achieved, and the yields of BPA and DMI increased to 96% and 98%, respectively. This phenomenon further proved that the addition of DMI does indeed have a promoting effect on the depolymerization of PC. Compared with previous work, although the ammonolysis of PC has been achieved by using DMEDA as a nucleophilic reagent and DMI as a solvent, in this work, the crucial role of the depolymerization product DMI in promoting the reaction was revealed for the first time [32].

### 2.5. Degradation of Common PC Waste Plastics

**Figure 6:**
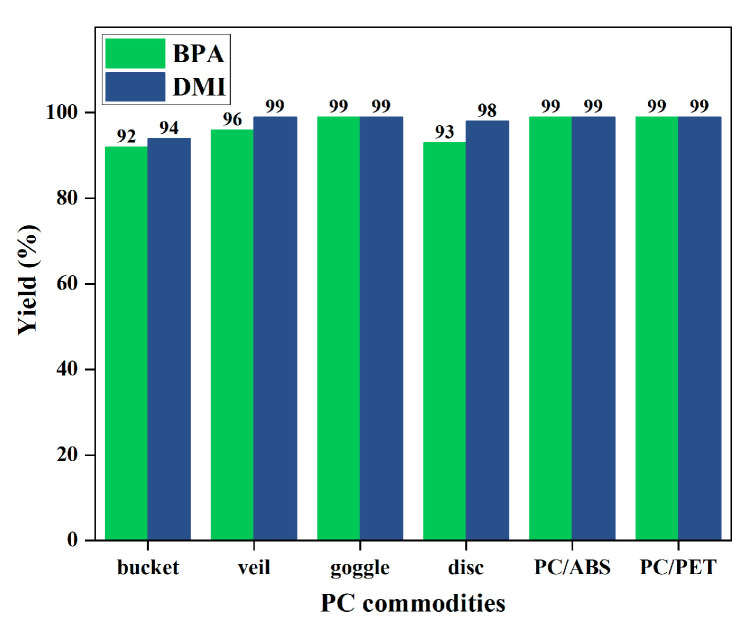
Degradation of PC commodities. Depolymerization conditions: PC commodities (254 mg, 1 mmol based on BPA unit), *N*,*N*′-dimethyl-1,2-ethanediamine.

**Figure 7:**
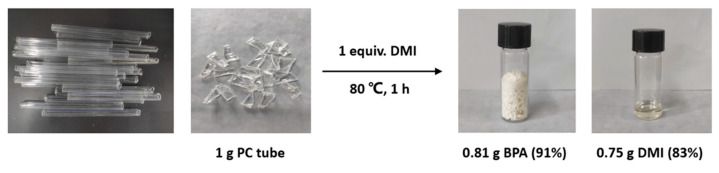
Depolymerization of PC tubes after consumption.

In order to demonstrate the efficiency and practicality of the product-promoted depolymerization strategy for PC materials, degradation experiments were conducted on common PC products and PC/ABS or PC/PET mixed plastics (Figure 6), such as PC buckets, face shields, goggles, and CDs, under optimized conditions (80 °C, 1 equiv. of DMI). The results showed that these PC products could be rapidly depolymerized and obtain depolymerized products with high yields. The corresponding BPA was obtained in 92–99% yields, and DMI was obtained in 94–99% yields. It is noteworthy that the molecular weights of these PC commodities (*M*_n_ = 12.7–19.2 kg mol^−1^) and the dispersities (*Đ* = 2.31–2.80) (Figure S5) varied, but all were suitable for this strategy. Furthermore, selective depolymerization of PC/ABS and PC/PET mixed plastics could also be achieved, providing BPA and DMI with >99% yields (Figure 6). These results offer a promising solution to address the challenges posed by plastic accumulation. Notably, chemical degradation of gram-scale PC tube material after consumption could also be successful in recovering high yields of BPA (91%) and DMI (83%) (Figure 7). These experimental results indicated that even with different additives present in commercial PC products, they have little or no impact on this depolymerization method, making this strategy highly applicable.

## 3. Materials and Methods

### 3.1. Materials and Reagents

The PC commodities (pellet, bucket, veil, goggle, disc, tube), polyethylene terephthalate (PET) bottle materials, and AcrylonitrileButadieneStyrene copolymer (ABS) granular material were purchased on Taobao.com or Amazon.cn and used without further purification. *N*,*N*′-dimethyl-ethylenediamine (DMEDA) (98%, Macklin, Shanghai, China), 1,3-dimethyl-2-imidazolidinone (DMI) (>99%, Aladdin, Shanghai, China), and bisphenol A (99%, Macklin) were used without further purification. Dibromomethane (98%, Energy Chemical, Shanghai, China) and hexamethylbenzene (98%, Energy Chemical) were used as internal standards. Dichloromethane (DCM) (>99%), tetrahydrofuran (THF) (>99%), ethyl acetate (EtOAc) (>99%), toluene (Tol.) (>99%), and acetone (Ace.) (>99%) were purchased as solvents at Sinopharm Chemical Reagent Co. (Shanghai, China). Chloroform-d (>99%) was purchased at Cambridge Isotope Laboratories, Inc. (Tewksbury, MA, USA). Dimethyl sulfoxide-d6 (>99%) and benzene-*d*_6_ (>99%) were purchased at Energy Chemical. Chromatographic THF was purchased from Honeywell LTD (Tokyo, Japan) for the analysis of GPC measurements.

### 3.2. Depolymerization of PC

The depolymerization of PC under solvent-free condition was carried out in a Schlenk flask at 80 °C. PC pellets (508 mg, 2 mmol based on BPA unit) were added first, followed by DMEDA (229 μL, 2.12 mmol). After a certain reaction time, dibromomethane (140 μL, 2 mmol) was added as the internal standard and acetic acid as the quencher. The reaction was monitored by ^1^H NMR spectrum. After 4 h, the yield of BPA was 73% and the yield of DMI was 74%. The depolymerization experiments of PC under the condition of DCM as a solvent were carried out at 50 °C, which was almost the same as the reaction conditions of the solvent-free depolymerization of PC mentioned above, and only an additional 2 mL of solvent was added.

Similarly, for the depolymerization of PC material under the condition of 1 equiv. of DMI, an additional 216 µL DMI was required on the basis of the solvent-free reaction condition. Furthermore, when degradation experiments were carried out with 1 g of PC commercial tube material under this condition, it was observed that the fragments of PC tube disappeared after 1 h. The product was separated by silica gel column chromatography, the developing agent was an n-hexane and ethyl acetate system, the volume ratio was 50–1:1, and then the products were vacuum-dried and weighed. After that, the yield was calculated.

### 3.3. The Determination of the Binding Constant

At room temperature, dry solid powder of BPA was dissolved in deuterobenzene (purified by vacuum distillation) to form a dilute solution of a certain concentration (0.0026 M), and 0.5 mL of it was loaded into a dry NMR tube to test its ^1^H NMR spectrum. Different equivalents of DMI (purified by vacuum distillation) were gradually dropped into the NMR tube using a micro-sampler, and their ^1^H NMR spectra were tested after shaking well. The ratio between the two substances was determined by integration of the ^1^H NMR spectra. The changes in the chemical shifts of the ^1^H NMR spectra after the interaction between the two substances were observed and recorded. The binding constant (K) was finally obtained by curve fitting in Origin [27,28].

### 3.4. Product Characterization

Nuclear magnetic resonance measurements were performed at room temperature on a Bruker Advance instrument at 400 MHz (^1^H NMR), 100 MHz (^13^C NMR), CDCl_3_ and (CD_3_)_2_SO were used as internal references. Molecular weight (*M*_n_) and dispersity (*Đ*) of the polymers were determined by gel permeation chromatography (GPC, Agilent 1260 LC, Santa Clara, CA, USA) using THF as the eluent (flow rate: 1 mL/min, at 40 °C). The chemical structures of the recovered products, BPA and DMI, were also confirmed by infrared spectroscopy and high-resolution mass spectrometry [33,34]. The FT-IR spectra were obtained on a Bruker Tensor 27 spectrophotometer. The high-resolution mass spectrometry tests were carried out on the Bruke Maxis UHR TOF instrument (Billerica, MA, USA).

## 4. Conclusions

In summary, a product-promoted depolymerization strategy for chemical recycling of PC was demonstrated. Using DMEDA as the depolymerization reagent, the reaction could be efficiently carried out under mild conditions with the promotion of the degradation product, DMI. The incorporation of 1 equiv. of DMI destroyed the hydrogen-bonding interactions between DMEDA and BPA, thus breaking the upper limit of the reaction and achieving a conversion of >95% for depolymerization. Different types of commercial PC plastics and PC/ABS or PC/PET mixed plastics, despite having different molecular weights and dispersities, were suitable for this strategy. This work is of great significance in promoting the chemical recycling of engineering plastic PC and provides a new idea and method for the sustainable use of plastic resources in the future.

## Figures and Tables

**Table 1:** The degradation of PC in different solvents.

Entry	Solvent	Yield of BPA (%)	Yield of DMI (%)
1	DCM	77	79
2	THF	68	66
3	EtOAc	75	75
4	Tol.	72	71
5	Ace.	70	68
6	DMI	87	85

Depolymerization conditions: PC pellets (508 mg, 2 mmol based on BPA unit), *N*,*N*′-dimethyl-1,2-ethanediamine (DMEDA) (229 μL, 2.12 mmol), hexamethylbenzene (64 mg, 0.4 mmol) as an internal standard to calculate yields, acetic acid as quencher, and V_solvent_ = 2 mL. Reaction at 50 °C for 24 h.

## Data Availability

Data are contained within the article and Supplementary Materials.

## Supplementary Materials

The following supporting information can be downloaded at: https://www.mdpi.com/article/10.3390/molecules29030640/s1, Figure S1: ^1^H NMR spectrum of PC degradation reaction under solvent-free conditions for 24 h (400 MHz, DMSO-*d*_6_, 298 K); Figure S2: Dissolution of 254 mg (1 mmol based on BPA unit) PC in 4 mL of different solvents at 50 °C; Figure S3: ^1^H NMR spectra of PC degradation in different solvents for 2 h (400 MHz, DMSO-*d*_6_, 298 K); Figure S4: Proposed mechanism; Figure S5: GPC analysis for PC disc, goggle, veil, and bucket; Figure S6: ^1^H NMR spectrum of BPA (400 MHz, DMSO-*d*_6_, 298 K); Figure S7: ^13^C NMR spectrum of BPA (400 MHz, DMSO-*d*_6_, 298 K); Figure S8: ^1^H NMR spectrum of DMI (400 MHz, DMSO-*d*_6_, 298 K); Figure S9: ^13^C NMR spectrum of DMI (400 MHz, DMSO-*d*_6_, 298 K); Figure S10: IR spectrum of BPA; Figure S11: IR spectrum of DMI; Figure S12: MS spectrum of BPA; Figure S13: MS spectrum of DMI; Table S1. Donor number values of common compounds.
