# Supplementary material for: Degradation Product-Promoted Depolymerization Strategy for Chemical Recycling of Poly(bisphenol A carbonate)
Source: Molecules. 2024 Jan 30;29(3):640. doi: 10.3390/molecules29030640 (PMC10856637; doi:10.3390/molecules29030640)
Supplement: Supplementary file 1 [file molecules-29-00640-s001.zip › molecules-2829968-supplementary.pdf]

Supporting Information

Degradation Product-Promoted Depolymerization Strategy for Chemical Recycling of Poly(Bisphenol A Carbonate)

Maoqing Chai [1,2], Guangqiang Xu [2,3,4,5,*], Rulin Yang [2,4,5], Hongguang Sun [1,*] and Qinggang Wang [2,3,4,5,*]

[1] College of Polymer Science and Engineering, Qingdao University of Science and Technology, Qingdao 266042, China; chaimq@qibebt.ac.cn
[2] Key Laboratory of Biobased Materials, Qingdao Institute of Bioenergy and Bioprocess Technology, Chinese Academy of Sciences, Qingdao 266101, China; yangrl@qibebt.ac.cn
[3] Center of Materials Science and Optoelectronics Engineering, University of Chinese Academy of Sciences, Beijing 100049, China
[4] Shandong Energy Institute, Qingdao 266101, China
[5] Qingdao New Energy Shandong Laboratory, Qingdao 266101, China
* Correspondence: xu_gq@qibebt.ac.cn (G.X.); hgsun816@qust.edu.cn (H.S.); wangqg@qibebt.ac.cn (Q.W.)

General procedure for the depolymerization of PC/PET and PC/ABS mixed plastics under the condition of addition of 1 equiv. DMI (Figure 6)

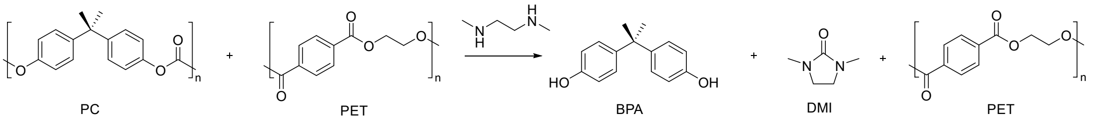

The depolymerization of PC and PET blends was carried out in a Schlenk flask at 80 °C. PC pellets (508 mg, 2 mmol, based on BPA units) and PET bottle small pieces (384 mg, 2 mmol, relative to the polymeric repeating unit) were added first, followed sequentially by N,N'-Dimethyl-1,2-ethylenediamine (DMEDA) (646 μl, 6 mmol) and 1,3-Dimethyl-2-imidazolidinone (DMI) (216 μl, 2 mmol). Dibromomethane (140 μL, 2 mmol) was added as the internal standard and acetic acid as the quencher. The reaction was monitored by ^{1}H NMR spectrum. After 3 h, the yields of BPA and 1, 3-dimethyl-2-imidazolidinone (DMI) were 99%.

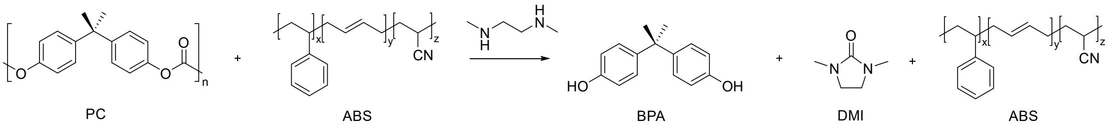

The depolymerization of PC/ABS alloy plastics (mass ratio 1:1) is carried out in a Schlenk flask at 80 °C. PC/ABS particles (254 mg, 0.5 mmol, based on BPA units) were added first, followed sequentially by N,N'-Dimethyl-1,2-ethylenediamine (DMEDA) (102 μl, 1 mmol) and 1,3-Dimethyl-2-imidazolidinone (DMI) (96 μl, 1 mmol). Dibromomethane (63 μL, 1 mmol) was added as the internal standard and acetic acid as the quencher. The reaction was monitored by ^{1}H NMR spectrum. After 3 h, the yields of BPA and 1, 3-dimethyl-2-imidazolidinone (DMI) were 99%.

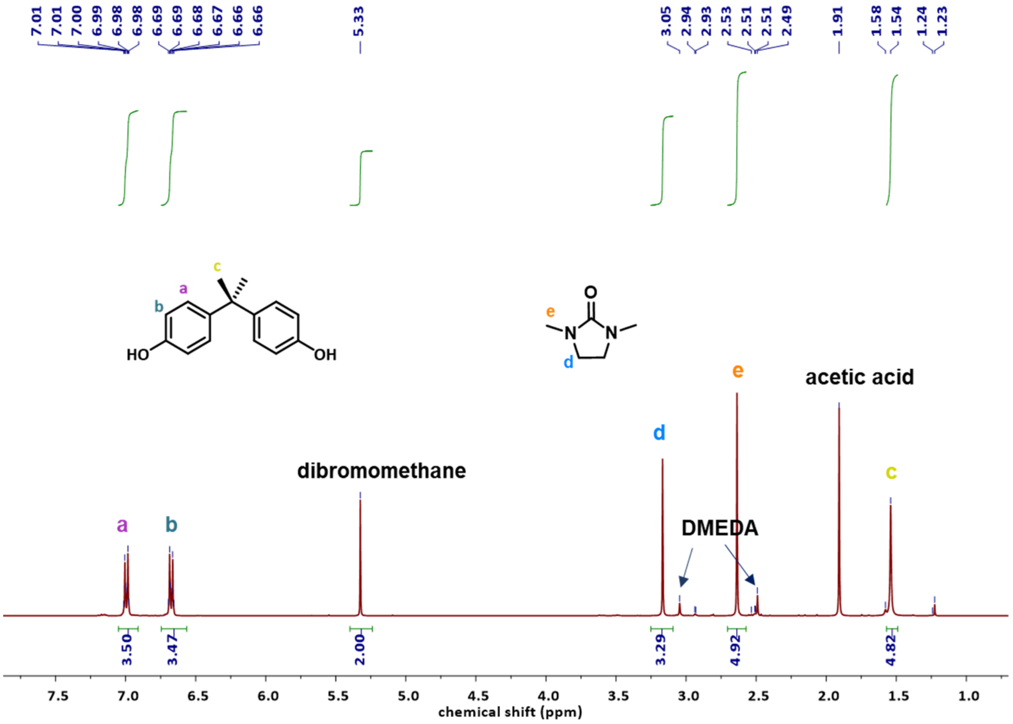

Figure S1. ^{1}H NMR spectrum of PC degradation reaction under solvent-free conditions for 24 h.

(400 MHz, DMSO-d_6, 298 K).

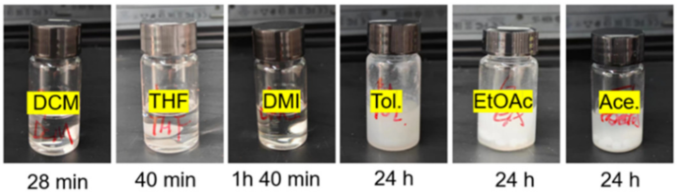

Figure S2. Dissolution of 254 mg (1 mmol based on BPA unit) PC in 4 mL of different solvents at 50 °C.

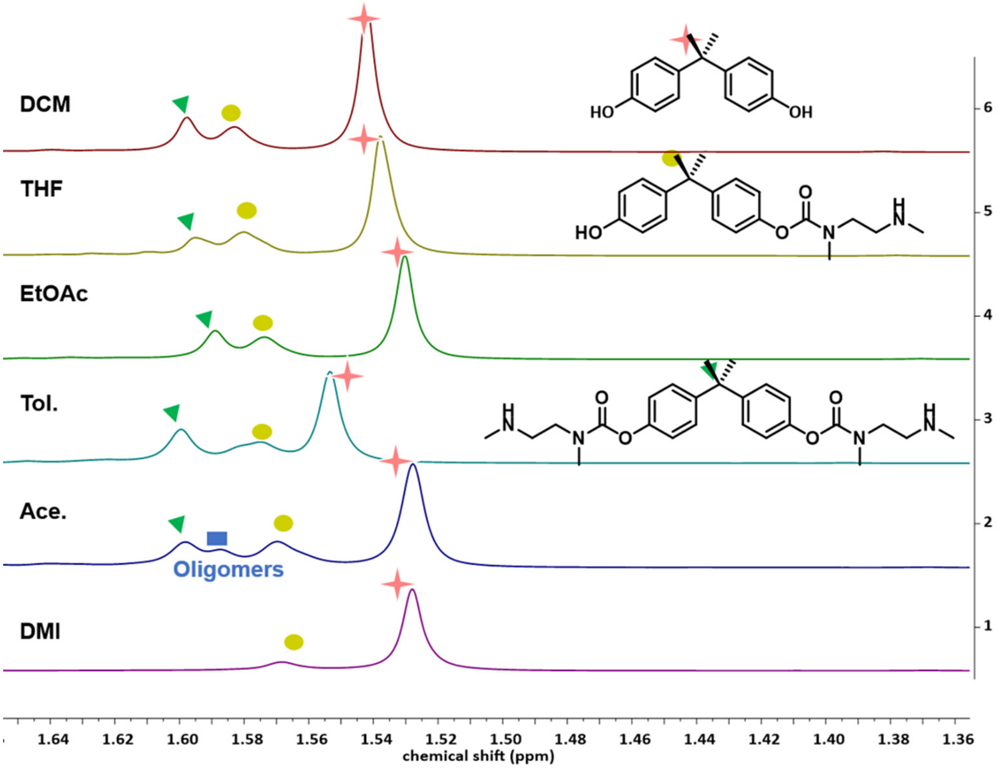

Figure S3. ^1H NMR spectra of PC degradation in different solvents for 2 h. (400 MHz, DMSO-d_6, 298 K).

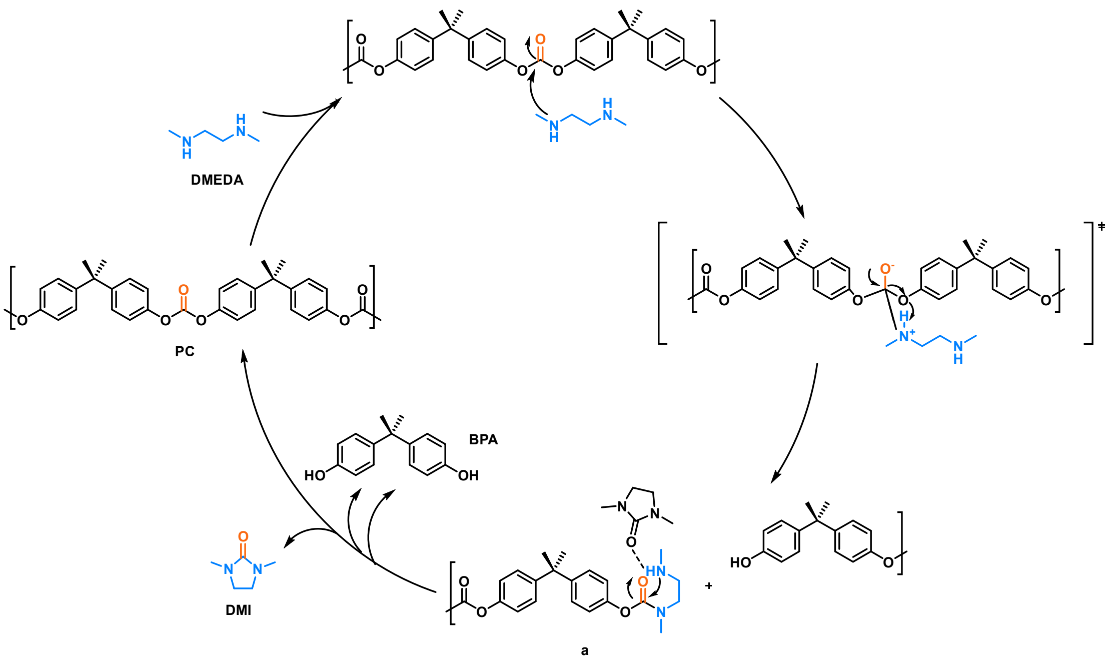

Figure S4. Proposed mechanism.

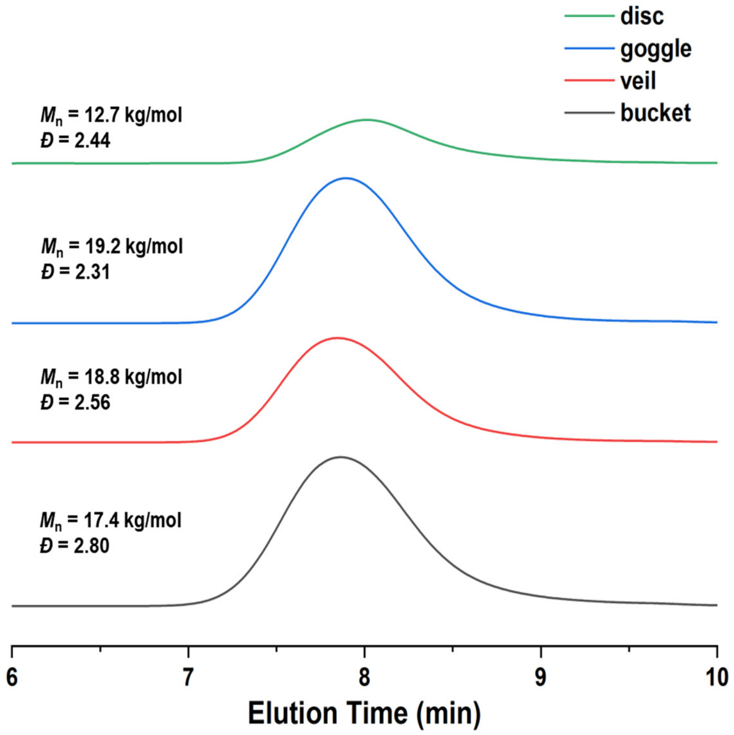

Figure S5. GPC analysis for PC disc, goggle, veil, and bucket.

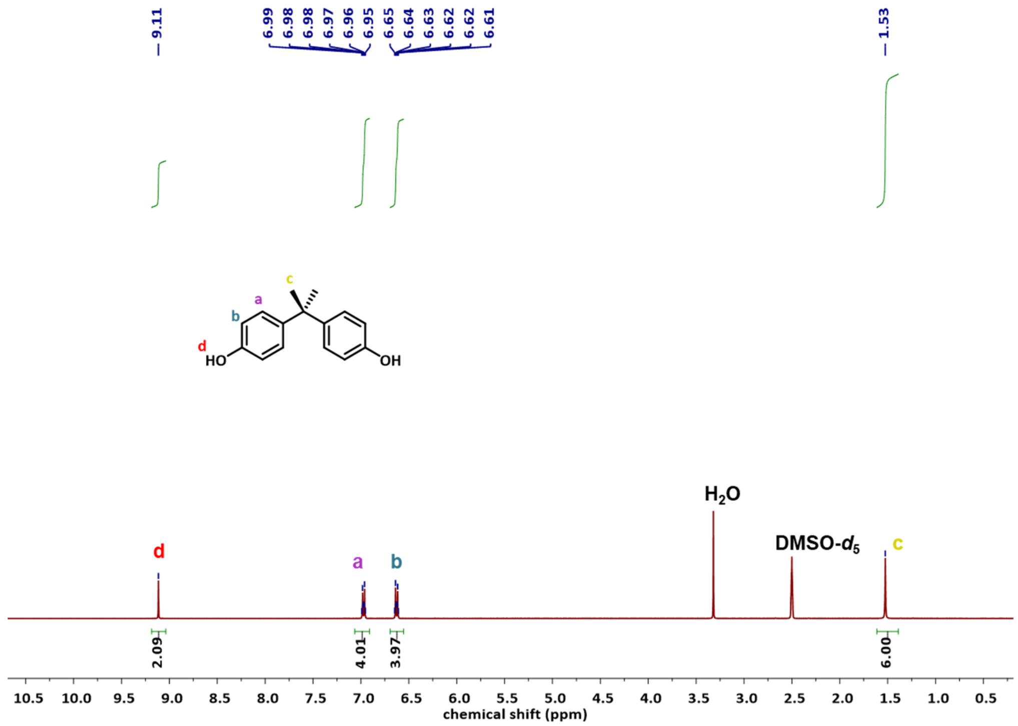

Figure S6. ^{1}H NMR spectrum of BPA (400 MHz, DMSO-d_6, 298 K).

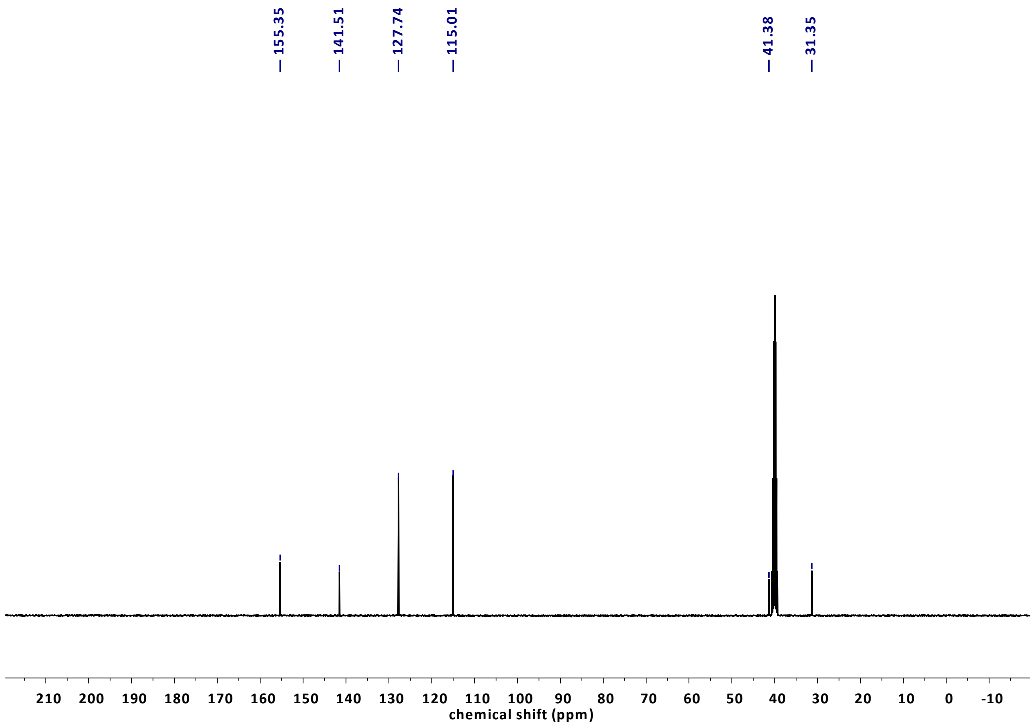

Figure S7. ^{13}C NMR spectrum of BPA. (400 MHz, DMSO-d_6, 298 K).

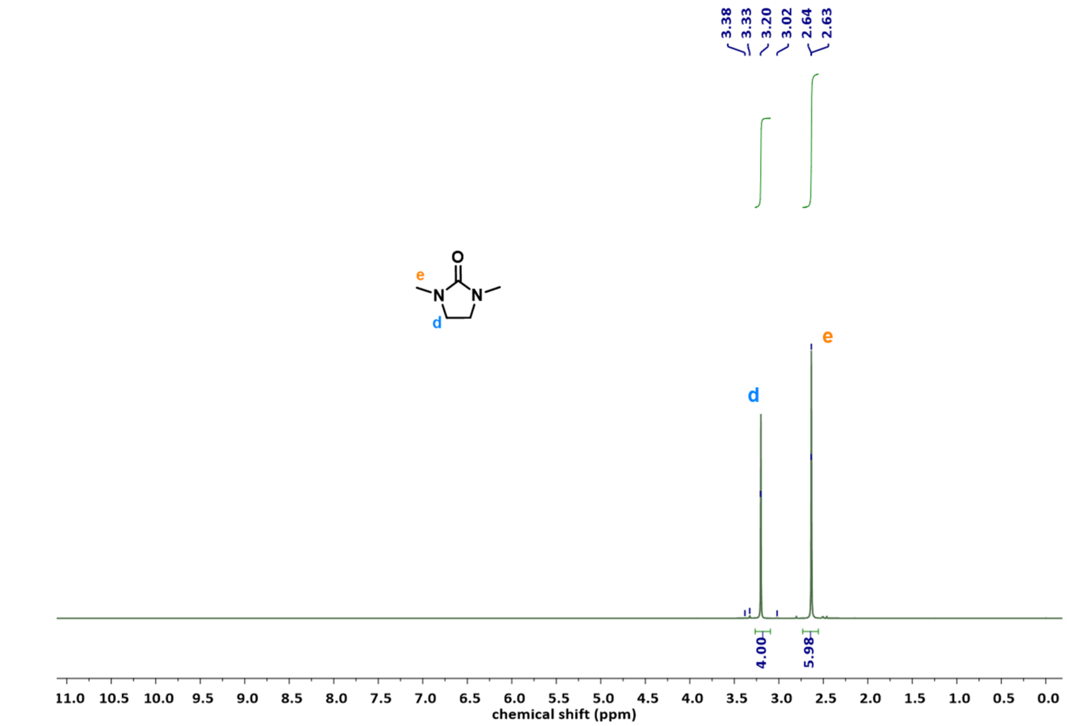

Figure S8. ^{1}H NMR spectrum of DMI. (400 MHz, DMSO-d_6, 298 K).

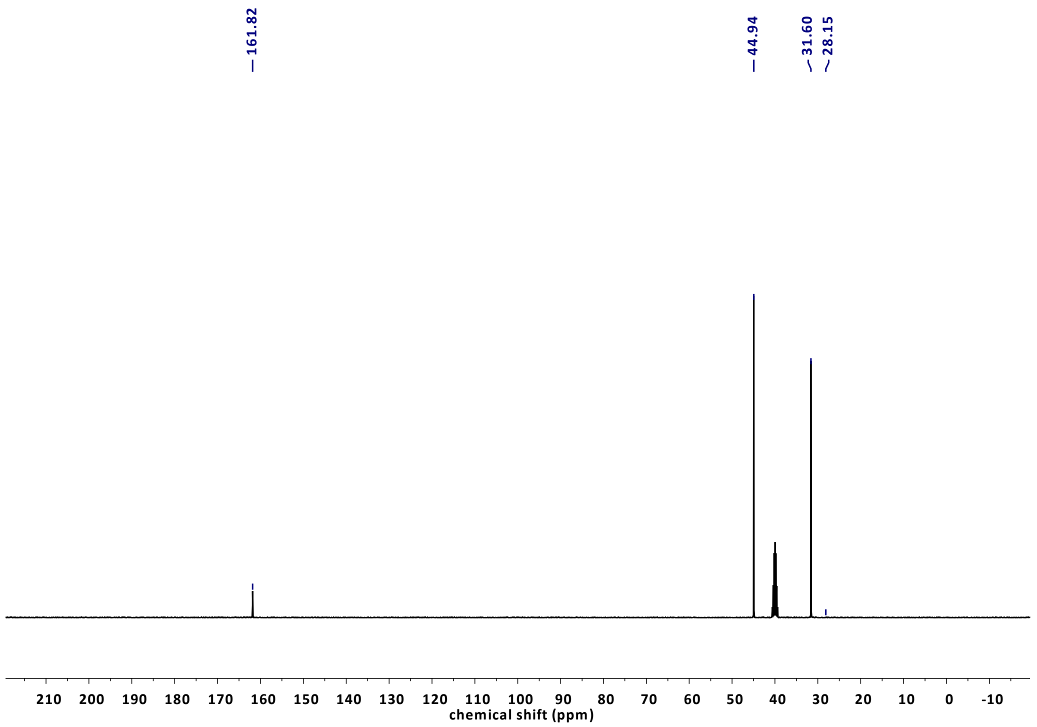

Figure S9. ^{13}C NMR spectrum of DMI. (400 MHz, DMSO-d_6, 298 K).

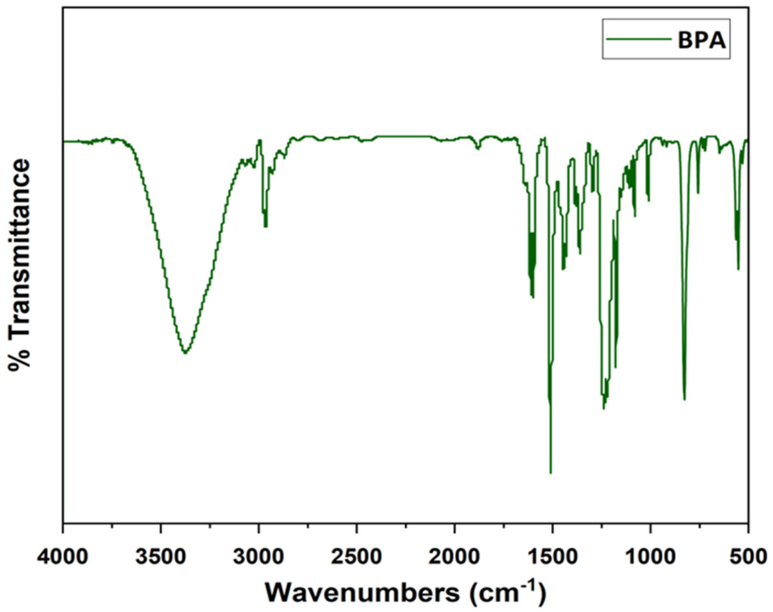

Figure S10. IR spectrum of BPA.

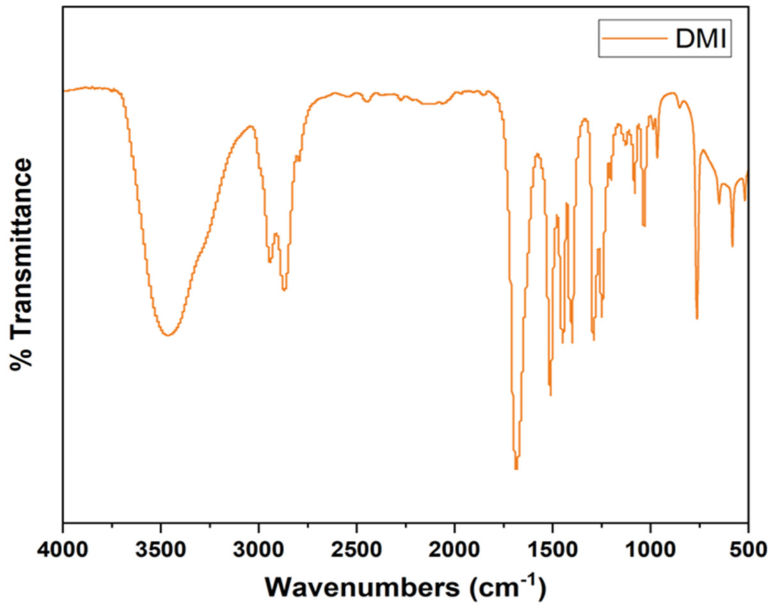

Figure S11. IR spectrum of DMI.

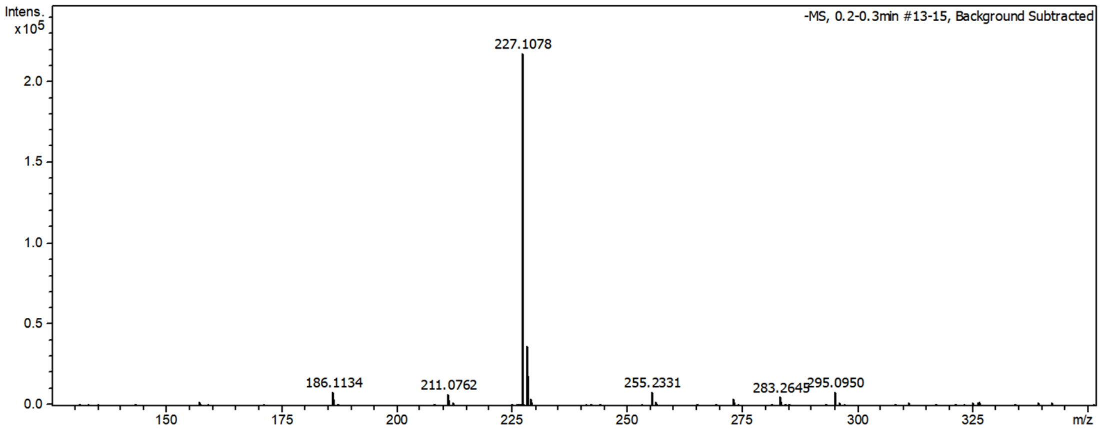

Figure S12. MS spectrum of BPA.

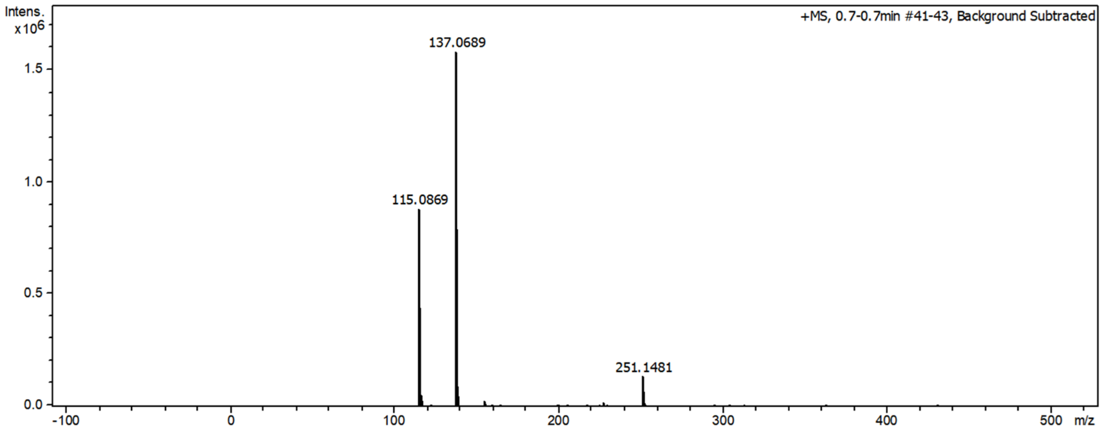

Figure S13. MS spectrum of DMI.

Table S1. Donor Number values of common compounds

Entry	Chemical Compound	Donor Number (kcal mol^{-1})
1	THF	21
2	DMI	29
3	EtOAc	16
4	Ace.	14
